# Electric field–dependent phonon spectrum and heat conduction in ferroelectrics

**DOI:** 10.1126/sciadv.add7194

**Published:** 2023-02-01

**Authors:** Brandi L. Wooten, Ryo Iguchi, Ping Tang, Joon Sang Kang, Ken-ichi Uchida, Gerrit E. W. Bauer, Joseph P. Heremans

**Affiliations:** ^1^ Department of Materials Science and Engineering, The Ohio State University, Columbus, OH 43210, USA.; ^2^ National Institute for Materials Science, Tsukuba 305-0047, Japan.; ^3^ Advanced Institute for Materials Research, Tohoku University, Sendai 980-8577, Japan.; ^4^ Department of Mechanical and Aerospace Engineering, The Ohio State University, Columbus, OH 43210, USA.; ^5^ Institute for Materials Research, Tohoku University, Sendai 980-8577, Japan.; ^6^ Center for Spintronics Research Network, Tohoku University, Sendai 980-8577, Japan.; ^7^ Zernike Institute for Advanced Materials, Groningen University, 9747 AG Groningen, Netherlands.; ^8^ Kavli Institute for Theoretical Sciences, University of the Chinese Academy of Sciences, Beijing 10090, China.; ^9^ Department of Physics, The Ohio State University, Columbus, OH 43210, USA.

## Abstract

This article shows experimentally that an external electric field affects the velocity of the longitudinal acoustic phonons (*v*_LA_), thermal conductivity (κ), and diffusivity (*D*) in a bulk lead zirconium titanate–based ferroelectric. Phonon conduction dominates κ, and the observations are due to changes in the phonon dispersion, not in the phonon scattering. This gives insight into the nature of the thermal fluctuations in ferroelectrics, namely, phonons labeled ferrons that carry heat and polarization. It also opens the way for phonon-based electrically driven all-solid-state heat switches, an enabling technology for solid-state heat engines. A quantitative theoretical model combining piezoelectric strain and phonon anharmonicity explains the field dependence of *v*_LA_, κ, and *D* without any adjustable parameters, thus connecting thermodynamic equilibrium properties with transport properties. The effect is four times larger than previously reported effects, which were ascribed to field-dependent scattering of phonons.

## Teaser

Lattice vibrations in ferroelectrics respond to external electric fields, giving a field-dependent thermal conductivity.

## INTRODUCTION

Magnetism and ferroelectricity, the ordered states of magnetic and electric dipoles in solids, are two phases of condensed matter that have much in common. Both orders can be very robust up to above room temperature and used in nonvolatile memories. The changes in entropy associated with both orders form the basis of magnetocaloric and electrocaloric technologies. Magnons, the quanta of the elementary excitations of the magnetic order, carry momentum, energy (*1*), and magnetization currents. By analogy, ferrons are introduced theoretically in (*2*, *3*) as the quanta of the elementary excitations of the electric polarization in ferroelectrics. The electric dipole *p_k_* (or electric polarization) of a single ferron with linear momentum *k* and energy dispersion ε*_k_* can be written in terms of a derivative with respect to an external electric field *E*, *p_k_* = − ∂ε*_k_*/*∂E* (*3*), just like the magnetization *m_k_* (or spin polarization) of a single magnon with linear momentum *k* and energy dispersion ε*_k_* can be written in terms of a derivative with respect to an external magnetic field. Here, as in (*2*, *3*), we use the operational definition of ferrons as being quasiparticles with finite electric polarization *p_k_* ≠ 0. Magnonics is presently one of the most active and highly sophisticated fields of magnetism, while an analogous field of “ferronics” does not exist. The concept of a ferron is to date a theoretical dream without any experimental confirmation.

Here, we present the first experimental evidence that the ferron exists by three independent measurements on the archetypal ferroelectric lead zirconium titanate (PZT). The experiments sample the acoustic phonons, so the condition *p_k_* ≠ 0 is equivalent to an electric field–dependent sound velocity. Previous theory predicts that in unbiased ferroelectrics, the polarization that governs electrocaloric effects are carried exclusively by the soft-mode optical phonons (*3*). However, here, we find that the symmetry breaking by piezoelectric strain polarizes the acoustical phonons by hybridization with the optical ones.

Ferrons are responsible for the decrease of the saturation polarization with increasing temperature. A temperature gradient applied to a ferroelectric therefore drives not only a heat flux *j_Q_* but also a polarization flux *j_P_* because the polarization at the hot side of the sample is lower than at the cold side. The latter is a nonequilibrium net flow of electric dipoles that should not be confused with the shift current in electrocaloric effects. The mixed transport of *j_P_* and *j_Q_* under applied effective electric field *E* and temperature *T* gradients obeys the Onsager relation (*2*)

$$\left(\begin{array}{c} -j_{P}\\ j_{Q} \end{array}\right)=\sigma \left(\begin{array}{cc} 1 & \text{Π}/T\\ \text{Π} & \kappa /\sigma \end{array}\right)\left(\begin{array}{c} \partial E\\ -\partial T \end{array}\right)$$ (1)

where Π is the polarization Peltier coefficient Π ≡ −*j_Q_*/*j_P_*∣_∇*T*=0_, while σ and κ are the polarization and thermal conductivity, respectively. The Onsager relation is macroscopic and does not depend on a specific microscopic mechanism.

**Fig. 1.**
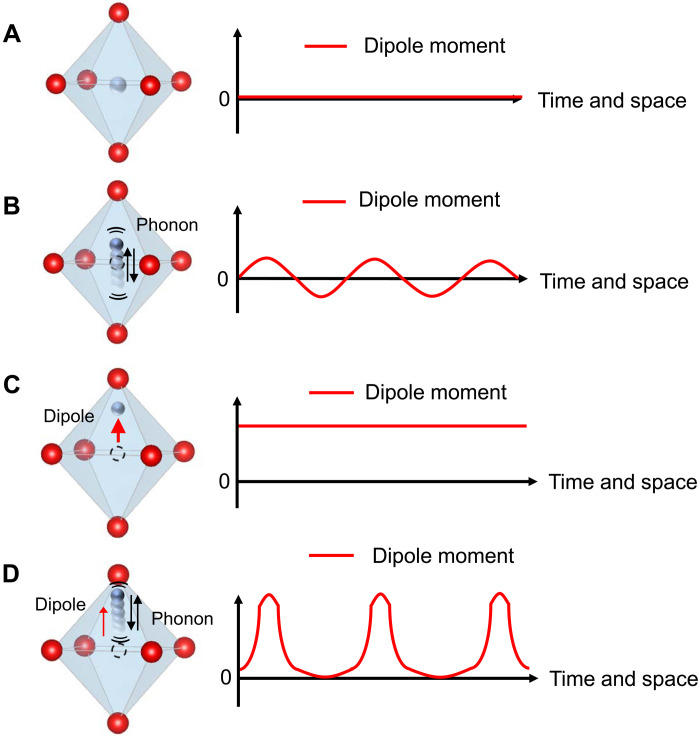
Schematic representation of a ferron in a displacive ferroelectric. An atomic representation and resulting dipole moment of a ferroelectric BaTiO_3_, where the red atoms are O^2−^ and the blue-gray atom is Ti^4+^. (**A**) The average atomic positions above the critical temperature *T_C_*. (**B**) A symmetric soft optical phonon excitation. (**C**) A polarized ferroelectric at *T < T_c_*, in which a net polarization (the red arrow) arises from a shift of the average position of the Ti ions. (**D**) A typical ferron excitation of a polarized ferroelectric. It reduces the net polarization because the ions oscillate in a nonlinear way around the equilibrium position (dashed red line).

Predictive microscopic theories, however, depend on the material and the nature of the ferroelectricity. In the majority of known ferroelectrics, the polarization is associated with the physical motion of charged ions or molecules, e.g., phonons. Bauer *et al*. (*2*) considered a one-dimensional (1D) order/disorder ferroelectric with rigid elementary dipoles and named the phonons involved in the polarization flux ferrons. In that model, the polarization Peltier coefficient is related to the susceptibility χ*_E_* of the material by χ*_E_* ≡ *dP*_0_/*dE*∣_∇*T*=0_ = *k*_B_*T*/*a*^3^Π^2^, where *P*_0_ is the equilibrium polarization and *a* is the lattice constant. Also in that model, the electric field–dependent thermal conductivity depends on *E* as κ = κ_0_(1 + *E*/Π). The concept of polarization-carrying phonons was extended to displacive ferroelectrics (*3*), such as BaTiO_3_, focusing on the polarization arising from the motion of titanium atoms vis-à-vis the oxygen octahedral cage. Figure 1 (A and B) illustrates the average ion position and the optical phonon above the ordering temperature *T_C_*. In a polarized material below *T_C_*, an ion in these solids is slightly displaced from its equilibrium position (Fig. 1C). The restoring force then becomes asymmetric around the equilibrium position of the atoms. Figure 1D illustrates very schematically the atomic motion associated with the anharmonic polar phonon in a polarized ferroelectric, a ferron. Such a ferron transports polarization because the ions oscillate in a nonlinear way about the equilibrium position (*3*), which reduces the equilibrium dipole moment. For large effects, such phonons should be very anharmonic. In displacive ferroelectrics, the polar phonon modes are optical, while acoustic modes dominate the heat and polarization transport because they have much higher group velocities. Here, we find that an electric field mixes the optical and acoustic modes by strain, leading to a field-dependent sound velocity and transport properties. This study uses PZT, in which the polarization involves the motion of the Pb atoms (*4*), which is difficult to visualize schematically; the precise phonons involved in the polarization in PbTiO_3_ are described in (*4*).

We report the measured temperature and electric field–dependent thermal conductivity κ(*E*,*T*), thermal diffusivity *D*(*E*,*T*) ≡ κ/*C* (*C* is the volumetric specific heat), longitudinal acoustic phonon velocity *v*_LA_(*E*) at 290 K, and susceptibility χ*_E_*(*E*,*T*) of a piezoelectric actuator stack made from bulk PZT with interdigitated electrodes. We provide a coherent and quantitative model that explains the logarithmic field dependences κ′*/*κ*, D*′*/D*, and *v*′*/v*, where κ′ ≡ *∂*κ*/∂E*∣*_T_*, *D*′ ≡ *∂D*/*∂E*∣*_T_*, and *v*′ ≡ *∂v*/*∂E*∣*_T_*. In this model, the electric field affects the phonon dispersion by piezoelectric strain (quantified by the piezoelectric moduli *d*_33_ and *d*_31_) and anharmonicity (quantified by the Grüneisen parameters), which leads to a change in the thermal transport properties. The model explains the data very well without invoking any adjustable parameters and extends the theory in (*3*) that does not consider either electrostriction or piezoelectric strain components.

This is of more than academic interest. Over 70% of the energy humanity uses comes from thermal sources (*5*), so devices that actively and dynamically control heat are technologically important (*6*). Heat switches control, direct, or pause heat flow in a system. They enable the creation of thermal rectifiers, thermal transistors, heat controllers, and more. They can increase the thermodynamic efficiency of entire classes of heat engines, particularly when these operate under time-dependent heat loads, such as solar-thermal systems (*6*). Classical heat engines thermodynamically process a fluid through various states in an entropy and temperature diagram. Heat switches enable all-solid-state thermodynamic cycles without moving parts using a medium (e.g., an electrocaloric or magnetocaloric solid) that exchanges heat for work. For example, in a Carnot cycle, heat switches would open during the isentropic processes and close during the isothermal ones when the medium is in contact with the hot or cold reservoirs. Existing heat switches control heat conduction mechanically through physical contact or the pumping of exchange gases, which are subject to wear and cyclic fatigue. Heat switches based on phase change materials work only at fixed temperatures, are slow, and suffer from thermal cycling problems. Mechanisms of all-solid-state heat switches, such as magnetic-field control of the electronic (*7*), spintronic (*8*), and magnonic (*1*, *9*) thermal conductivity, have other drawbacks, such as low operating temperatures and a lattice thermal conductivity that limits the ratio of the conductance in the on and off states. Efficient electrical field control of κ(*E*) has been reported in thin films exposed to electrolytes (*10*). An all-solid-state switch operating over a wide temperature range, including at higher temperatures, would be technologically very attractive. This paper describes one of the few mechanisms based on external control of the lattice thermal conductivity that have ever been proposed or found. We report a four to five times larger effect of the electric field on κ(*E*) at room temperature compared to previous work (*11*, *12*). The fundamental understanding of the physical principles for an electric field–actuated phonon-based heat switch demonstrated here is a first step to engineer more efficient devices.

## RESULTS

**Fig. 2.**
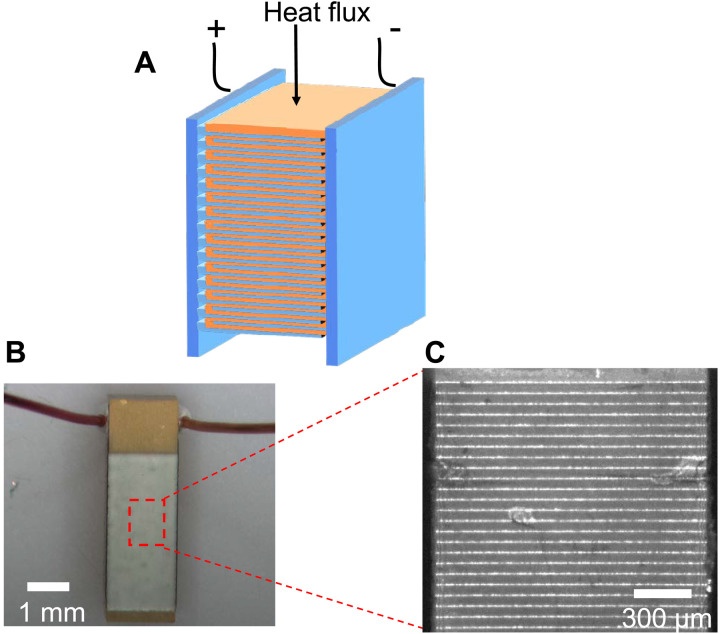
Piezoelectric stack. (**A**) Schematic diagram and (**B**) picture; the dimensions of the unencapsulated stack, gray in the picture, are 1.65 mm by 1.65 mm by 3.69 mm. (**C**) A micrograph showing the electrodes (bright) and PZT material (dark) of the PZT-based actuator used in experiments. The micrograph was taken with an infrared camera to emphasize the contrast. The distance between the interdigitated electrodes is 48 μm.

**Fig. 3.**
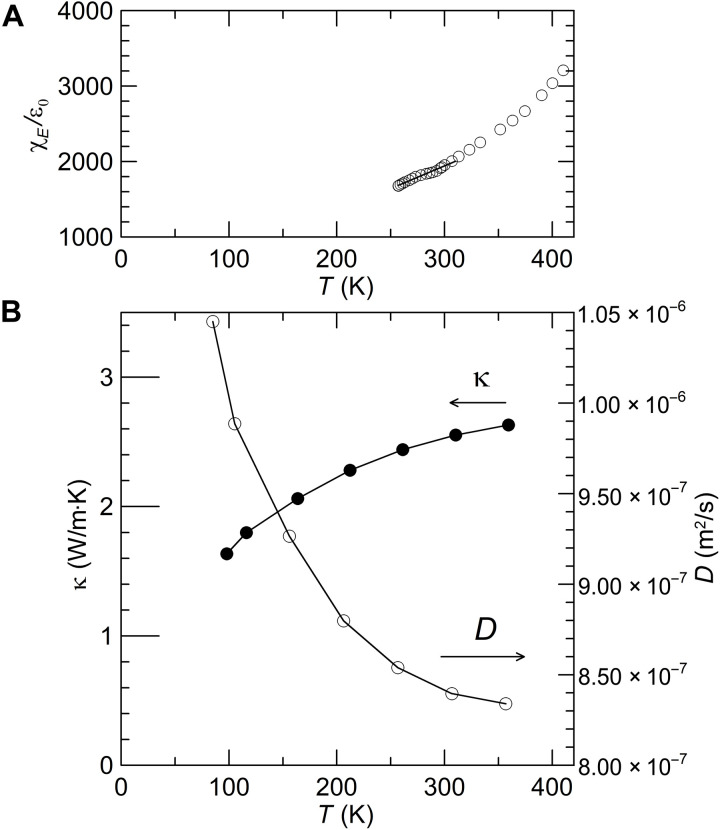
Temperature dependence of zero-field properties of unpolarized PZT stack. (**A**) Relative permittivity χ*_E_*(*E* = 0,*T*) normalized to the permittivity of vacuum, ε_0_. (**B**) Thermal conductivity κ(*E* = 0,*T*) and thermal diffusivity *D*(*E* = 0,*T*) of the unpolarized PZT stack.

A PZT-based actuator with interwoven silver electrodes was obtained commercially (TailKuke, no. 603126) with a working voltage of 100 V. The PZT had an approximate composition of Pb[Ti_0.37_Zr_0.24_Nb_0.25_Ni_0.14_]O_3_ (see Materials and Methods). Figure 2A shows a schematic of the digitated silver electrodes (blue) and the PZT ceramic (orange). The electric field is positive when aligned with the polarization. In each layer of one pair of interdigitated electrodes, both the field and the polarization alternate polarity, so the field keeps the same polarity throughout the stack. Figure 2B shows a picture of the device, which is encapsulated in polymers. Figure 2C shows a grayscale picture of the metal electrodes (white) and PZT layers (gray). Figure 3A reports the low-field susceptibility χ*_E_*(*E* = 0*,T*) of the unpolarized device. Figure 3B displays the thermal conductivity measured using a static heater-and-sink method. The measurements cover temperatures from 97 to 359 K and are corrected for the silver electrode contribution and heat loss during the experiment. The interfacial thermal resistance between the electrodes and the PZT material in the stack was measured separately using the time-domain thermoreflectance (TDTR) method and is shown to increase the total thermal resistance by no more than 1.3%. The value of κ is about half that of PbTiO_3_ at 350 K (*4*). This and the fact that the slope *d*κ*/dT* of the ceramic alloy studied here is positive, while that of PbTiO_3_ is negative (*4*), indicate that alloy scattering of the heat-carrying phonons dominates here. Because our equipment did not allow us to make a measurement of the heat capacity as a function of electric field, we measured the thermal diffusivity of the samples directly using the Ångström method (*13*). The value at zero applied electric field is also reported in Fig. 3B. Details of the measurement techniques are given in Materials and Methods.

**Fig. 4.**
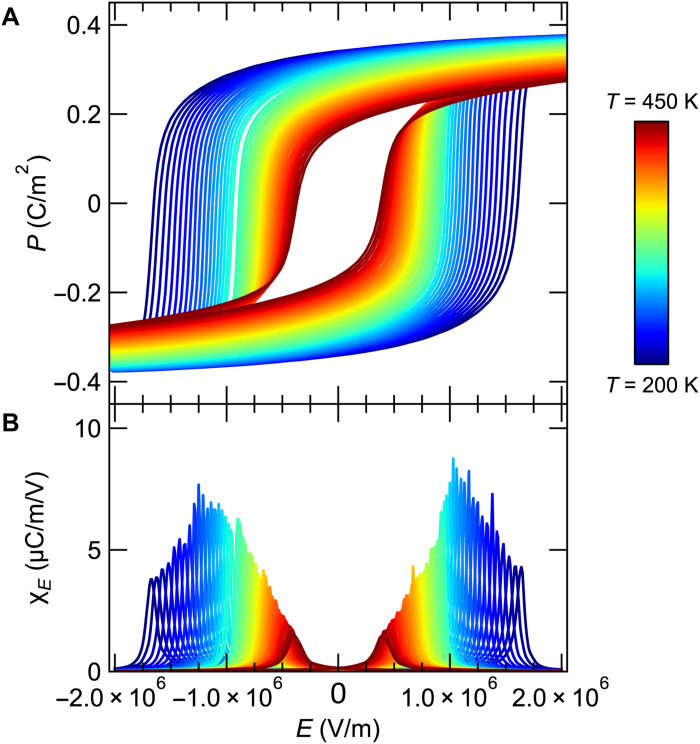
Electric field dependence of polarization and permittivity. (**A**) Polarization *P*(*E*,*T*) and (**B**) permittivity χ*_E_*(*E*,*T*) of the PZT sample as a function of the electric field *E*. The coercive field gradually increases as the temperature decreases.

**Fig. 5.**
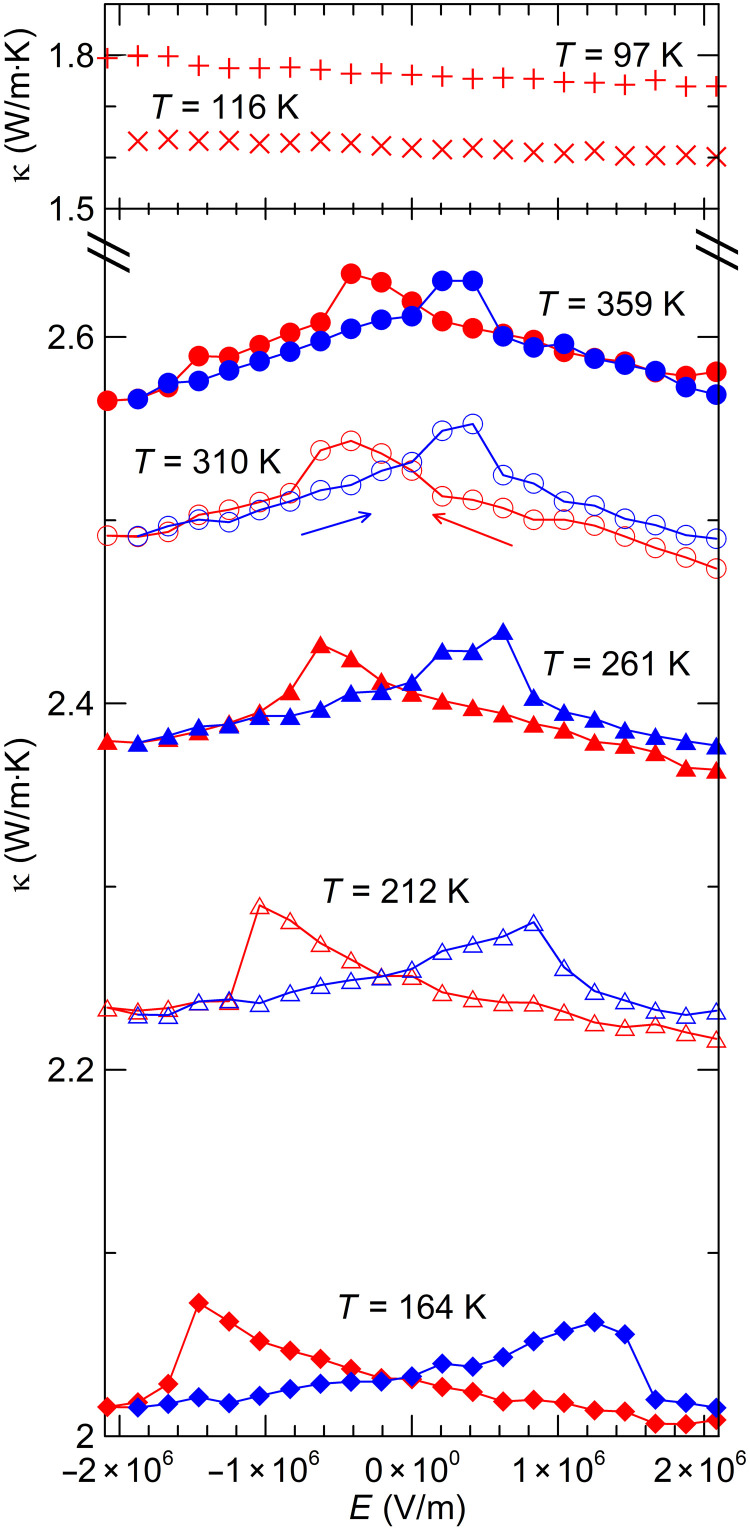
Electric field dependence of the thermal conductivity κ(*E*,*T*) through the hysteresis loops. The coercive field exceeds the maximum field that could safely be applied to the sample at *T* < 160 K so that only one branch of the hysteresis loop is shown at 116 K and below, which still allows for the determination of κ′/κ.

**Fig. 6.**
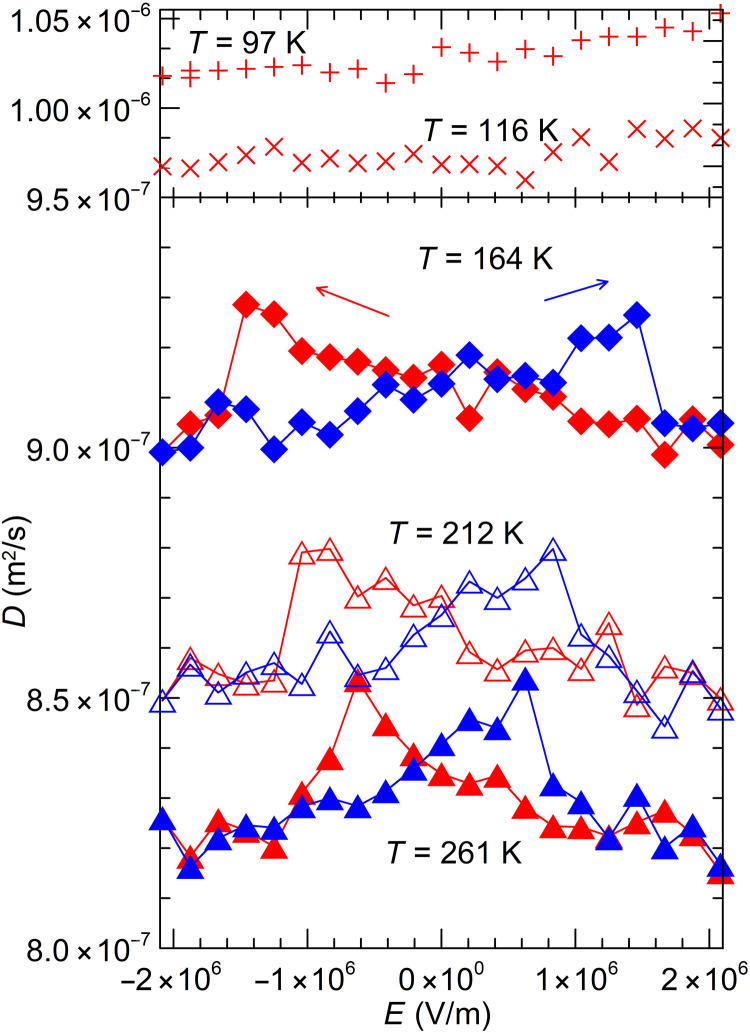
Electric field dependence of the thermal diffusivity *D*(*E*,*T*) through the hysteresis loops. As in Fig. 5, the hysteresis loop could not be completed at 116 and 97 K, but *D*′*/D* is still measurable.

Focusing now on the electric field dependence of these quantities, Fig. 4A gives the polarization as a function of electric field and temperature measured using a Sawyer-Tower circuit (*14*). The polarization was found to saturate at *P*_sat_ = 0.2 C m^−3^, and the coercive field was about *E_C_* = 0.5 × 10^6^ V/m at room temperature. *E_C_* increases with decreasing temperature as expected. The isothermal susceptibility χ*_E_* ≡ *∂P*/*∂E*∣*_T_* is shown in Fig. 4B. Figure 5 reports the thermal conductivity κ(*E*,*T*) as a function of electric field at various temperatures, measured using a static heater-and-sink method (see Materials and Methods). The hysteresis loops could not be completed at lower temperatures: The saturation polarization is reached only at *T* > 160 K. Below that temperature, only one branch can be measured but is sufficient to determine κ′ ≡ ∂κ/*∂E*∣*_T_*. Figure 6 reports the same properties for the diffusivity *D*(*E*,*T*), measured directly on the stack using the Ångström method. As for the thermal conductivity, the coercive field was not reached at *T* < 150 K. In this case, the sample remained polarized such that the values of *E* reported in the figure are antiparallel to *P* (i.e., the abscissa is really −*E*) so that *D*′ < 0.

**Fig. 7.**
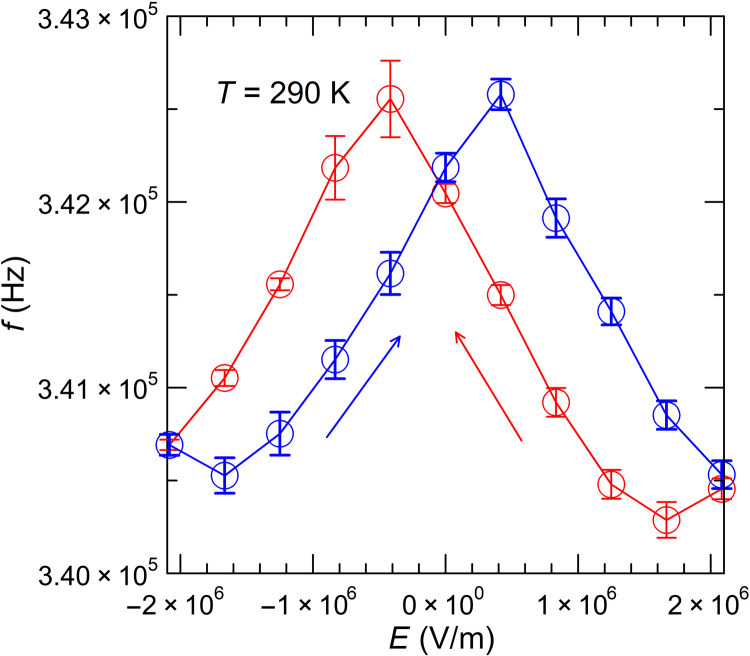
Electric field dependence at 290 K of the ultrasound resonance around 340 kHz. About 340 kHz corresponds to the longitudinal compressive wave through the sample and, thus, to the longitudinal acoustic sound velocity *v*_LA_.

Using resonant ultrasound spectroscopy (RUS) (*15*), we further measure the longitudinal acoustic phonon sound velocity. Figure 7 shows the electric field dependence of the mechanical resonance frequency *f*_L_ of the longitudinal compressive mode of the stack at room temperature. This resonance is identified as 80% because of the elastic constant *c*_11_ of the PZT along the polarization direction (see Materials and Methods). From it, we derive a sound velocity *v*_LA_ ≈ 3.2 km/s to within 10% (see Materials and Methods). Note that because *f*_L_ ∝ *v*_LA_, *f*_L_′*/f*_L_ *= v*_LA_′*/v*_LA_.

**Fig. 8.**
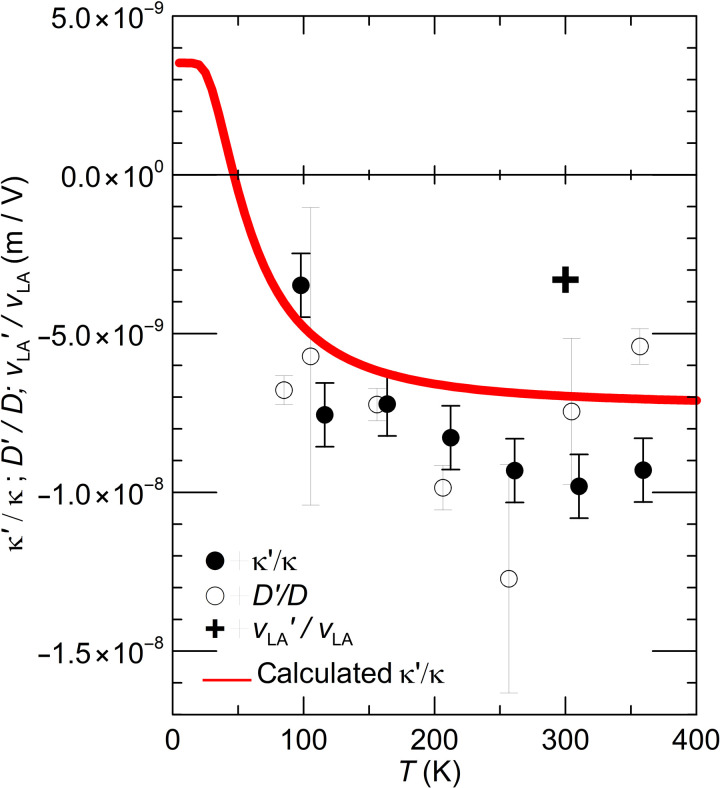
Temperature dependence of the logarithmic derivatives. Temperature dependence of the logarithmic derivatives of the thermal conductivity κ′*/*κ (full dots), thermal diffusivity *D*′*/D* (open circles), and sound velocity *v*_LA_′*/v*_LA_ (cross) of the PZT stack vis-à-vis electric field. The calculated values of κ′*/*κ are shown as a red line. A sign change is predicted in the calculated κ′*/*κ because it scales with *v*′/*v* above.

To calculate the logarithmic derivatives κ′*/*κ, *D*′*/D*, and *f*_L_′*/f*_L_ *= v*_LA_′*/v*_LA_, linear regressions were taken on each quantity and each temperature between the electric field where the quantity is maximum to the maximum field. The average slopes of these regressions gave κ′, *D*′, and *f*_L_′. The values of κ, *D*, and *f*_L_ were taken at zero field. The results are shown in Fig. 8. As noted in the introduction, κ′*/*κ at 300 K is a factor of ~5 larger than in (*11*). The error bars in Fig. 8 combine the relative errors in the field dependences of the quantities and the errors in the regression slopes.

## DISCUSSION

Alloy disorder scattering of phonons dominates the thermal conductivity, but we assume that this mechanism does not depend on the electric field. Below, we discuss in detail the possibility that phonon scattering on ferroelectric domain walls, whose size depends on the applied electric field, might be responsible for the observation but rule it out on the basis that near–room temperature phonon-phonon Umklapp scattering results in phonon mean free paths that are much shorter than the domain size in bulk samples. Instead, we develop a model based solely on the modification of the acoustic phonon dispersion caused by field-induced elastic deformation. In ferroelectric materials, the reverse piezoelectric effect generates a strain within the crystal lattice, and the displacement of ions under an electric field, in turn, changes the phonon dispersion, sound velocities, and thermal conductivity and diffusivity. Moreover, Tong *et al.* (*16*) points out that strain-induced changes in the phonon spectrum contribute to the electrocaloric effect.

The strain tensor component *e*_33_ gives the compression/expansion along the direction of the ferroelectric order, and components *e*_11_ = *e*_22_ give the deformations perpendicular to the ferroelectric order. They are related to the electric field by the piezoelectric coefficients *d*_33_ and *d*_31_ by

$$d_{33}=\frac{\partial e_{33}}{\partial E};d_{31}=\frac{\partial e_{11}}{\partial E}$$ (2)

The relative volume *V* change of a sample is expressed as a function of the strain tensor components by δ*V*/*V* = *e*_11_ + *e*_22_ + *e*_33_. This changes with electric field as

$$\frac{d\ln V}{dE}=d_{33}+2d_{31}$$ (3)

Using the label λ = (LA, TA) to denote the mode and ignoring crystalline anisotropy, the phonon frequencies ω_λ_(*k*) *= v*_λ_*k* at low momentum *k* now depend on the electric field to the first order in momentum *k* as

$$\frac{d\omega_{\lambda }(k)}{dE}=\frac{d\omega_{\lambda }(k)}{dV}\frac{\partial V}{\partial E}=-\gamma_{\lambda }(d_{33}+2d_{31})\omega_{\lambda }(k)+O(k^{2})$$ (4)

γ_λ_ are the low-frequency mode Grüneisen parameters $\gamma_{\lambda }\equiv -d\ln\omega_{\lambda }(k)/d\ln V+O(k)$. Consequently, in this model

$$\frac{{v_{\lambda }}^{\prime }}{v_{\lambda }}=\frac{1}{v_{\lambda }}\frac{dv_{\lambda }}{dE}=-\gamma_{\lambda }(d_{33}+2d_{31})$$ (5)

Assuming a Debye model and defining the mode-averaged phonon velocity as ${\bar{v}}^{-3}=(v_{\mathrm{LA}}^{-3}+2v_{\mathrm{TA}}^{-3})/3$, we have

$$C=\frac{3k_{B}^{4}T^{3}}{2\pi^{2}\hbar^{3}{\bar{v}}^{3}}\int_{0}^{\text{Θ}/T}\frac{x^{4}e^{x}}{{\left(e^{x}-1\right)}^{2}}dx$$ (6)

and κ is expressed in the Boltzmann formalism by

$$\kappa =\frac{\tau k_{B}^{4}T^{3}}{6\pi^{2}\hbar^{3}}\left(\frac{1}{v_{\mathrm{LA}}}+\frac{2}{v_{\mathrm{TA}}}\right)\int_{0}^{\text{Θ}/T}\frac{x^{4}e^{x}}{{\left(e^{x}-1\right)}^{2}}dx$$ (7)

The mode- and direction-averaged value of the Debye temperature Θ depends on $\bar{v}$ as $\text{Θ}=\hbar \bar{v}/k_{B}\sqrt[{3}]{6\pi^{2}N/V}$, where *N* is the number of unit cells in volume *V*. The field derivative of the thermal conductivity is

$$\frac{d\kappa }{dE}=\frac{d\kappa }{d\text{Θ}}\frac{d\text{Θ}}{dE}+\sum_{\lambda }\frac{d\kappa }{dv_{\lambda }}\frac{dv_{\lambda }}{dE}$$ (8)

and similarly for *dC/dE*. That of the diffusivity is

$$\frac{dD}{dE}=\sum_{\lambda }\frac{dD}{dv_{\lambda }}\frac{dv_{\lambda }}{dE}$$ (9)

The use of a Debye model is not a priori well justified because low-lying optical modes are present in the phonon spectrum (*4*), but the model works unexpectedly well, presumably because the optical modes have a very low group velocity. We now first test this model for consistency between κ′*/*κ, *D*′*/D*, and *f*_L_′*/f*_L_ *= v*_LA_′*/v*_LA_. Second, we compute these quantities at room temperature using only the values of the piezoelectric and Grüneisen parameters from the specification sheet of the material and the literature without adjustments. Third, we derive the temperature dependence of κ′*/*κ also without adjustable parameters.

First, at *T* > Θ and ignoring the optical modes, the specific heat reaches asymptotically the Dulong-Petit value and is constant. In that case

$$D\equiv \frac{\kappa }{C}\approx \frac{{\bar{v}}^{3}\tau }{9}\left(\frac{1}{v_{\mathrm{LA}}}+\frac{2}{v_{\mathrm{TA}}}\right)$$ (10)

Both κ and *D* become proportional to ${\bar{v}}^{2}$ so that, if the scattering time is not field dependent

$$\frac{\kappa^{\prime }}{\kappa }\approx \frac{D^{\prime }}{D}\approx 2\frac{{\bar{v}}^{\prime }}{\bar{v}},\mathrm{for}\;T>\text{Θ}$$ (11)

Furthermore, because the piezoelectric coefficients of the material do not depend on the mode and γ*_L_ =* γ*_T_* = 15 (*4*), ${\bar{v}}^{\prime }/\bar{v}=v_{LA}^{\prime }/v_{LA}=v_{TA}^{\prime }/v_{TA}$. For PbTiO_3_, Θ ~ 160 ± 30 K as calculated from the spectrum given in (*4*). Equation 10 applies at room temperature and provides a parameter-independent test for the model. Table 1 summarizes the room temperature experimental values for these quantities in Eq. 11, which is satisfied.

**Table 1.** Comparison of the experimental and calculated values. Comparison of the experimental and calculated values of the logarithmic derivatives of the sound velocity, thermal conductivity, and thermal diffusivity at room temperature. The agreement provides a parameter-independent test for the model and Eq. 11.

Quantity	Measurement (m/V)	Model (m/V)
*v*_L_*′/v*_L_ *= v*_T_*′/v*_T_	−3.3 ± 0.5 × 10^−9^	−3.5 × 10^−9^
κ*′/*κ	−9.5 ± 1.0 × 10^−9^	−7.0 × 10^−9^
*D′/D*	−7.2 ± 2.0 × 10^−9^	−7.1 × 10^−9^

Second, a quantitative comparison requires knowledge of the piezoelectric and Grüneisen parameters of the material. Only *d*_33_ = 435 × 10^−12^ m/V is provided by the manufacturer. Taking γ*_L_ =* γ*_T_* = 15 (*4*) and *d*_31_ = −100 pm/V (the *d*_31_ value used is that for PbTiO_3_) (*17*), it is possible to derive the values given in the column “model” in Table 1 from Eqs. 5 and 11. Again, they fit the measurements markedly well, particularly given the simplicity of the model and the fact that contributions by the optical phonons are disregarded.

Third, the model is extended to variable temperatures including *T* ~ Θ by solving Eq. 7 and considering the temperature dependence of the piezoelectric coefficients. The electrostriction coefficients *Q_ij_* relate the strain to the polarization *P*_0_ and are given by

$$e_{33}=Q_{11}P_{0}^{2};e_{11}=e_{22}=Q_{12}P_{0}^{2}$$ (12)

and the piezoelectric coefficients *d*_33_ and *d*_31_ can be written as

$$d_{33}=2Q_{11}P_{0}\chi_{E};d_{31}=2Q_{12}P_{0}\chi_{E}$$ (13)

For PbTiO_3_ with a centrosymmetric paraelectric parent phase, the Landau-Ginzburg-Devonshire (LGD) theory for displacive ferroelectrics (*18*, *19*) gives the polarization and susceptibility as

$$\begin{array}{rl} P_{0}^{2} & =\frac{-\alpha_{11}+\sqrt{\alpha_{11}^{2}-3\alpha_{1}\alpha_{111}}}{3\alpha_{111}}\\ \chi_{E} & =\frac{1}{2(\alpha_{1}+6\alpha_{11}P_{0}^{2}+15\alpha_{111}P_{0}^{2})} \end{array}$$ (14)

where α_1_, α_11_, and α_111_ are the Landau expansion coefficients. Furthermore, α_1_*=*α_0_ (*T − T*_C_) where α_0_ is the Curie-Weiss constant and *T*_C_ is the ferroelectric Curie temperature. For constant *Q*_11_ and *Q*_12_, *d*_33_ and *d*_31_ increase with temperature (note that we disregard here the small effect of the thermal expansion on the Debye temperature). The calculated temperature-dependent κ′*/*κ for PbTiO_3_ using the LGD parameters that are given in (*20*) is about half that of the κ′*/*κ measured. The difference is due to *d*_33_, which is 340 × 10^−12^ m/V in PbTiO_3_ versus 435 × 10^−12^ m/V in the PZT studied. Adjusting the calculated κ′*/*κ using the room temperature value of *d*_33_ = 435 × 10^−12^ m/V, we obtain the red curve in Fig. 8, which reproduces the temperature-dependent data. Note that for *T* < < Θ, the specific heat scales as (*T/*Θ)^3^, and therefore, $\kappa^{\prime }/\kappa \approx -{\bar{v}}^{\prime }/\bar{v}$ so that a change of sign is predicted to occur in κ′*/*κ at around 40 K.

These conclusions can be compared to previous results in the literature. Mante and Volger (*21*) found that κ(*E*) in single-crystal BaTiO_3_ increases two- to fivefold at *T* = 5 K by applying fields of *E =* 1.1 × 10^6^ V/m. This result was interpreted in terms of phonon scattering by ferroelectric domain walls. At *T* < 15 K, domain wall scattering supersedes phonon-phonon Umklapp scattering so that the thermal conductivity increases as domains grow under the applied field. However, this mechanism is not expected to work near room temperature, where the Umklapp-dominated phonon mean free path is much shorter than the domain size or in samples in which alloy scattering of phonons does the same. The phonon mean free path of the samples studied here can be roughly estimated from the diffusivity and sound velocity data to be $\frac{3D}{v_{\mathrm{LA}}}∼1\;\mathrm{nm}$ at 100 K, whereas in bulk PZTs near the morphotropic phase boundary, the domain size is of the order of 100 nm (*22*).

Ihlefeld *et al.* (*11*) report a decrease of [κ(*E*)−κ(*E =* 0)]/κ(*E =* 0) ~ −11% for *E =* 4.14 × 10^7^ V/m or κ′*/*κ *= −*2.6 × 10^−9^ m/V in PZT thin films at room temperature, roughly a factor of 4 smaller than the results as reported here. The authors attribute the decrease in κ(*E*) to an increasing domain wall density with increasing *E*, a counterintuitive idea but well supported by piezoresponse force microscopy (*23*) and scanning electron microscopy data (*24*). Recently, Aryana *et al.* (*12*) report [κ(*E*)−κ(*E =* 0)]/κ(*E =* 0) ~ −10% for *E =* 6 × 10^7^ V/m (κ′*/*κ *= −*1.7 × 10^−9^ m/V) in antiferroelectric PZT of a different composition than the ferroelectric material used here. The change of κ with *E* is interpreted as originating from grain boundary scattering induced by the presence of both antiferroelectric to ferroelectric phases and their phase change at *E =* 3.7 × 10^7^ V/m. Here, we report a two to five times larger effect with κ′*/*κ *= −*5 × 10^−9^ m/V at 100 K and reaching κ′*/*κ *= −*1 × 10^−8^ m/V at *T* > 300 K in bulk polycrystalline PZT samples.

In summary, we observe an electric field dependence of the thermal conductivity of a bulk ferroelectric PZT from 97 to 359 K, almost five times larger in magnitude to that reported in the literature (*11*, *12*). The present data on bulk samples near room temperature can be explained by piezoelectric strain that mixes the polarization of the optical modes into the acoustic ones. The good correspondence between the values obtained for the electric field derivatives of the sound velocity, a property at thermodynamic equilibrium, and the thermal conductivity and diffusivity, which are transport properties, corroborates this theory, arguing that this is not a scattering effect but a property of the phonon spectra.

To generalize these findings, Eq. 5 offers insight on how to select materials that maximize electric field–induced lattice thermal conductivity changes. Two properties are necessary: (i) high Grüneisen parameters indicating highly anharmonic acoustic phonons and (ii) a high piezoelectric coefficient. To maximize κ′*/*κ, it is also useful to minimize κ, here not only by alloy disorder scattering but also by applying other techniques inspired from research on thermoelectrics, such as nanostructuring (*25*). The field-dependent sound velocity implies that the piezoelectric strain mixes the optical phonons sketched in Fig. 1, which dominate the electrocaloric properties with the acoustic phonons. Ferrons can thus be both optical and acoustic phonons.

## MATERIALS AND METHODS

### Sample preparation and characterization

We investigate a commercial P5-8Y PZT–based piezoelectric actuator stack with a maximum working voltage of 100 V (Fig. 1, A to C). Manufacturer specifications include a mechanical quality factor *Qm* = 90 and a piezoelectric coupling coefficient *d*_33_ = 435 × 10^−12^ m/V. Chemical analysis by inductively coupled plasma gives a composition of the PZT to be Pb[Ti_0.37_Zr_0.24_Nb_0.25_Ni_0.14_]O_3_ with an uncertainty of ±11% on the stoichiometry. The data reported here were reproduced on other samples (see figs. S1 and S2). An infrared transmission spectrum of the P5-8Y material is shown in the supplement [fig. S3 and (*26*)] The stack consists of ~60 interdigitated Ag-Pd contacts, each sandwiching a layer of PZT about 48 μm thick. The width and thickness of the stack were both 1.65 mm. The polymer coatings on the stack were removed before mounting.

### Measurements

#### Polarization

The polarization *P* versus the electric field *E* curves were measured on the basis of the Sawyer-Tower method (*14*), where the polarization change of the system under periodic *E* is monitored through accumulated charges in a reference capacitor connected in series. An ac voltage of ~104 V at 1.0 Hz was applied to the PZT sample and reference capacitor of 10 μF in series. The time-dependent voltage in the reference capacitor was measured using a data acquisition device (NI, NI-9215) and converted into *P*(*t*), taking into account the sample dimensions and parasitic capacitance of connected cables. To measure the temperature (*T*) dependence of the *P-E* curve, the sample mounted on a sapphire substrate using insulating varnish (General Electronics, GE 7031) was placed in a cryostat for *T* < 300 K at vacuum and on a Peltier device for *T* > 300 K at atmospheric pressure. In the cryostat, the temperature of the sample was first lowered to 200 K without applying *E* (i.e., not poled) and increased after measurements at each temperature step. The derivative ∂*P/*∂*E* in Fig. 4B was obtained from the spline interpolation of the *P-E* curve.

#### Thermal conductivity

The thermal conductivity κ was measured using a static heater-and-sink method with a nitrogen-cooled Janis cryostat. The sample was mounted atop an alumina base acting as the heat sink (fig. S4). Two thermocouples consisting of a Constantan and a copper wire were attached to the side of the PZT actuator using GE varnish and their voltages measured using two Keithley K2182a nano-voltmeters. A resistive heater of 120 ohms on top of the stack generated temperature gradients for a minimum of 20 min for stability. Applied voltages were allowed to settle for 5 min before measurements were taken to suppress pyroelectric artifacts. The linearity of Fourier’s law was checked using several heater powers, and deviation from a linear trend was less than 0.1%. To assess heat loss in the cryostat at temperatures of 200 to 400 K, the thermal conductivity of a rod of electrolytic iron was measured and compared to National Institute for Science and Technology standard calibration values (*27*). The difference between the measured conductance and the value calculated of the iron sample from the calibration tables determined the amount of heat lost, which was subtracted from the total heat input into the PZT sample at each temperature point above 200 K in the experiments with the PZT actuator. Because the PZT stack consists of alternating layers of Ag and PZT, its thermal resistance consists of the sum of the resistances of the PZT layers, the Ag layers, and the interface contact resistances, acting like thermal resistances connected in series. However, because the thermal conductivity of Ag (~420 W/m·K) is two orders of magnitude larger than that of this PZT alloy (~2 W/m·K), it is possible to account for the contribution of the Ag layers by correcting the length of the sample for the relative thickness of the Ag and PZT alloy layers.

The thermal conductivity κ(*E* = 0,*T*) of the unpoled material is derived from the measurements and is reported in Fig. 2B. The values correspond well with the literature (*28*). The error in absolute value of the thermal conductivity is dominated by the geometrical error on the measurement of the distance between the thermocouples and is of the order of 5%. The relative error of the temperature dependence is due to inaccuracies of the correction for heat losses, of the order of 5% at 359 K, but less than 1% at 97 K. The relative error on the field dependence is dominated by the signal-to-noise ratio on the thermocouples typically <0.2%.

#### Interfacial thermal resistance measurement by TDTR

Because the stack consists of about 60 PZT layers and contains, thus, 120 electrode/PZT interfaces, the possibility that the interfacial contact resistance could contribute substantially to the total thermal resistance was investigated. The interfacial thermal conductance between PZT and the Ag electrode was measured directly at room temperature using the TDTR method, a reliable and standard method with an accuracy of about 10% for interfacial thermal conductance measurements. We used Ag for the transducer because it was already used as the electrode in the PZT stack. The Ag electrode thickness in the PZT actuator is 14 μm, which is too thick to yield a good sensitivity of the measurement to the interfacial thermal conductance between the transducer and PZT layers. To solve this issue, in our measurement, we prepared one PZT actuator and polished one of its electrodes down to form a silver wedge at about a 1.6° angle. The laser spot was chosen on that wedge at a point where the thickness of the Ag layer was down to ~100 nm; the exact thickness of the Ag was derived from the geometry and the optically measured planar dimensions of the Ag layer. We investigated the sensitivity of the TDTR measurement to the Ag thermal conductivity, to the interfacial thermal resistance between Ag and PZT, and to thermal conductivity of PZT, respectively (fig. S7), and found it to be sufficient to resolve the interfacial thermal conductance. The measured interfacial conductance value is 6 × 10^6^ W/m^2^∙K at each interface. The interfacial thermal resistance thus contributes only 1.3% to the total thermal resistance of the PZT layers alone and, consequently, is neglected here.

#### Thermal diffusivity

The thermal diffusivity *D* was measured using the Ångström method (*13*) on the same sample with the same mounting procedure as the thermal conductivity (fig. S5A). The heat in the resistive heater was varied sinusoidally following *Q* = *Q*_0_ cos (ω*t*) at a frequency of ω/2π = 0.24 Hz along the length of the PZT stack. That frequency was selected so that the thermal diffusion length (see below) in the sample is of the order of 1 to 1.5 mm, comparable to the distance between the thermocouples and the distance between the hot thermocouple and the heater. The voltage across the two thermocouples, measured with two K2182a nano-voltmeters, was recorded for 3000 cycles. An example of the collected data is shown in fig. S5 (B to E). Software was written to emulate the operation of a lock-in amplifier on the two thermocouple signals, giving the phase ϕ and amplitude |*T*| of the temperatures *T*_HOT_ and *T*_COLD_ at two points along the length of the sample distant *x*_HOT_ and *x*_COLD_ from the heater. To find the relationship between the phase, amplitude, and the diffusivity, a 1D semi-infinite rod model was used, with varying heat applied at point *x* = 0. The temperature as a function of both length *x* along the rod and time *t* is

$$T(x,t)=T_{0}e^{-kx}\cos(\omega t-kx-\phi )$$ (15)

where $k=\sqrt{\omega /2D}$ is the propagation vector, *D* the diffusivity, and ϕ an instrumental phase shift. The thermal diffusion length is 1/*k*. Taking a measurement of temperature at two locations *x*_HOT_ and *x*_COLD_ on the PZT stack, the propagation vector *k* and thus *D* can be derived from the amplitude attenuation [ln(*T*_HOT_*/T*_COLD_)] or from the phase shift

$$k=\frac{\ln|T_{\mathrm{HOT}}|-\ln|T_{\mathrm{COLD}}|}{x_{\mathrm{HOT}}-x_{\mathrm{COLD}}}=\frac{\varphi_{\mathrm{HOT}}-\varphi_{\mathrm{COLD}}}{x_{\mathrm{HOT}}-x_{\mathrm{COLD}}}$$ (16)

The amplitude attenuation gives better accuracy, while the phase is used to check for experimental consistency. The relative accuracy of this method is of the order of 1%, compared to a relative error of typically just under 10% when using TDTR but not as good as the error on the thermal conductivity change, which is <0.2%.

#### Sound velocity

We obtained an estimate for the longitudinal acoustic sound velocity of the sample at room temperature using a RUS (*15*) instrument (Alamo Creek Engineering, Santa Fe, NM, USA), while voltage was applied using a Data Precision 8200. In the RUS instrument, a parallelepiped sample is placed between two piezoelectric actuators, and the mechanical resonances were measured (see fig. S6). The electrical contacts to the sample were replaced by long and coiled 25-μm-diameter wires to perturb the natural resonances as little as possible. In a rectangular isotropic (polycrystalline) sample, the frequency (*f*_L_) of the fundamental longitudinal vibration mode is related to the effective Young’s modulus. The sample here is a Ag/PZT multilayer composite, but the Young’s modulus of Ag (69 to 74 GPa) and of PZT material [70 to 79 GPa with the voltage contacts open-circuit (*29*)] are quite close to each other, so it is possible to consider an effective medium approach for this mode and interpret *f*_L_ in terms of a single effective Young’s modulus *E_Y_* using the relation

$$E_{Y}=4\rho L^{2}f_{L}^{2}/K$$ (17)

where ρ = 7893 kg/m^3^ is the measured density and *L* = 4.23 mm is the measured length. *K* is a correction factor that is a function of the dimensions of the sample and the Poisson ratio, about 0.34 (*30*), here, *K* = 0.95. The measured value of *f*_L_ = 342.5 kHz gives a Young’s modulus of 69.7 GPa. The Young’s modulus in isotropic solids is $E_{Y}=c_{11}[1-2{\left(c_{12}/c_{11}\right)}^{2}+O{\left(c_{12}/c_{11}\right)}^{3}]$, where *c_ij_* are the components of the elastic constant tensor. In PbTiO_3_,, *c*_12_*/c*_11_ ~ 0.3 (*30*), and *E_Y_* is within 20% of *c*_11_. Because the longitudinal acoustic wave sound velocity $v_{\mathrm{LA}}=\sqrt{c_{11}/\rho }$, *f*_L_ ∝ *v*_LA_ and a longitudinal acoustic sound velocity can be derived to be $v_{\mathrm{LA}}\approx \sqrt{E_{Y}/\rho }$ = 3.2 km/s, with an uncertainty on the absolute value of 10%. The relative error on the logarithmic derivative is much smaller and due to instrument noise. It is given as the error bar in Fig. 7. The same simplifications do not apply to the shear modes, whose properties are not determined from the collected RUS spectrum. (*27*).

## Notes

View/request a protocol for this paper from *Bio-protocol*.
