# Supplementary material for: Electric field–dependent phonon spectrum and heat conduction in ferroelectrics
Source: Sci Adv. 2023 Feb 1;9(5):eadd7194. doi: 10.1126/sciadv.add7194 (PMC9891688; doi:10.1126/sciadv.add7194)
Supplement: Supplementary file 1 — Figs. S1 to S7 [file sciadv.add7194_sm.pdf]

Science Advances
AAAS

Supplementary Materials for

Electric field–dependent phonon spectrum and heat conduction in ferroelectrics

Brandi L. Wooten *et al.*

Corresponding author: Joseph P. Heremans, heremans.1@osu.edu

Sci. Adv. **9**, eadd7194 (2023)
DOI: 10.1126/sciadv.add7194

This PDF file includes:

Figs. S1 to S7

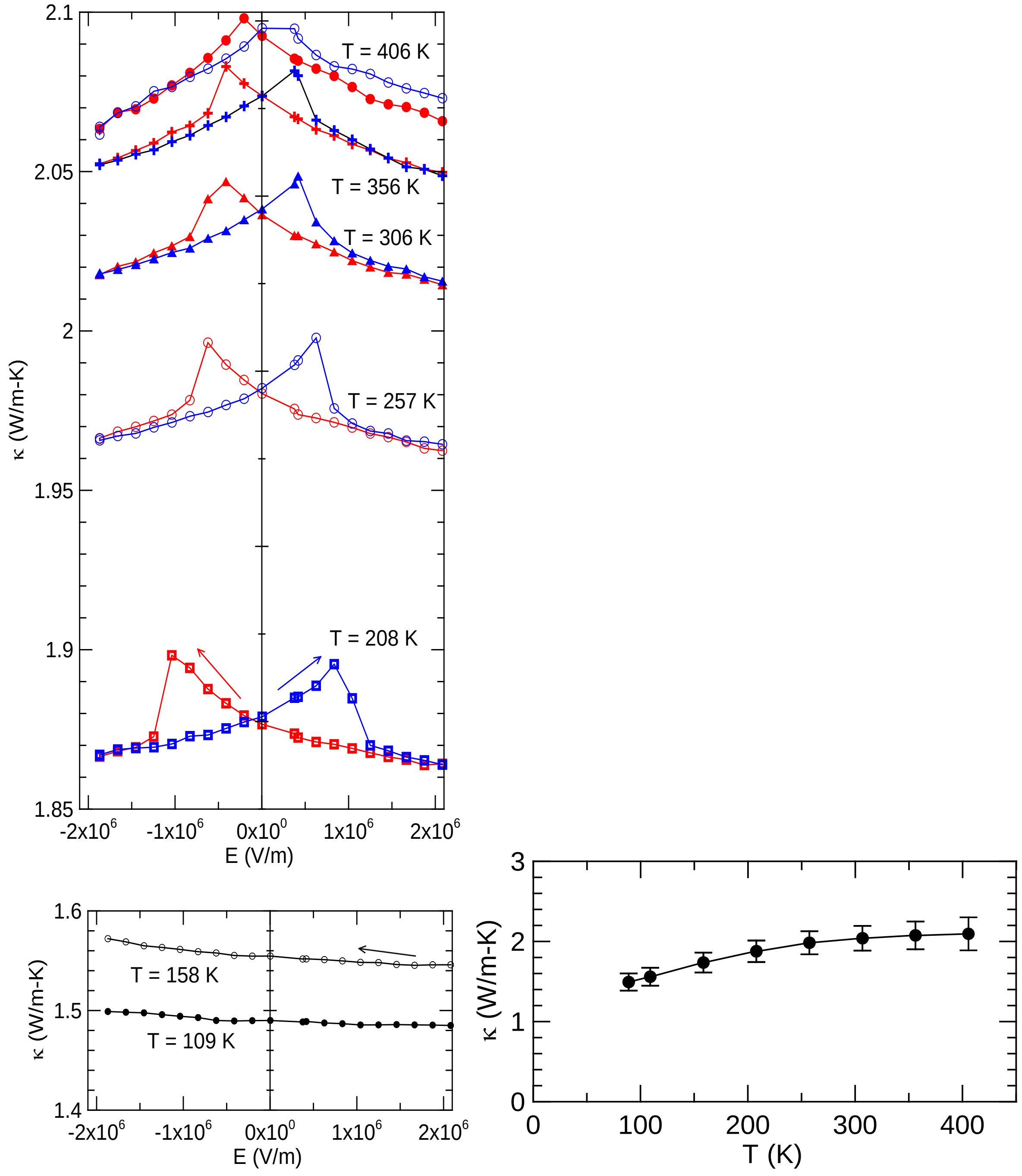

Fig. S1. Electric field and temperature dependence of thermal conductivity on second sample. The electric field dependence sweeps through the hysteresis loops. The coercive field exceeds the maximum field that could safely be applied to the sample at $T < 160$ K, so that only one branch of the hysteresis loop is shown at 158 K and below. This figure illustrates the sample-to-sample reproducibility of the results given in the main text.

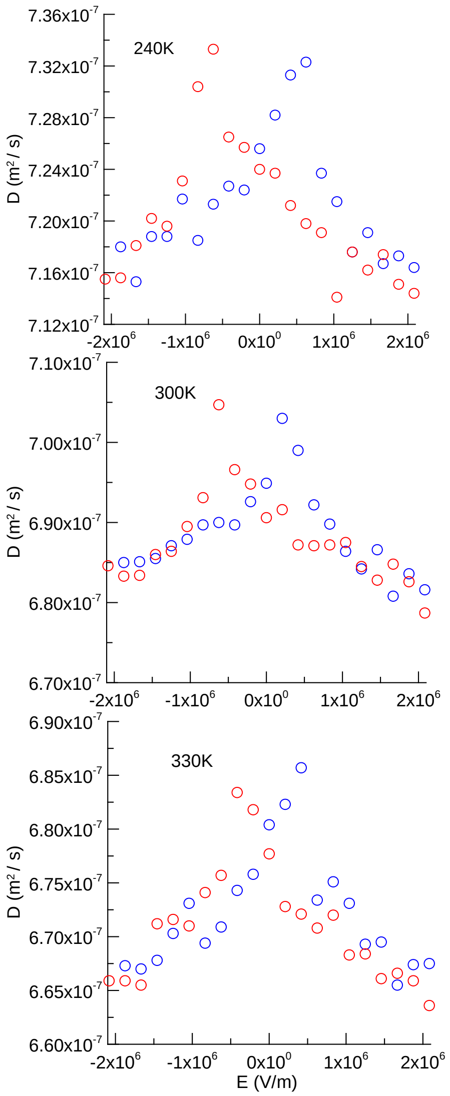

Fig. S2. Electric field dependence of thermal diffusivity at several temperatures of a third sample. This figure illustrates the sample-to-sample reproducibility of the results given in the main text.

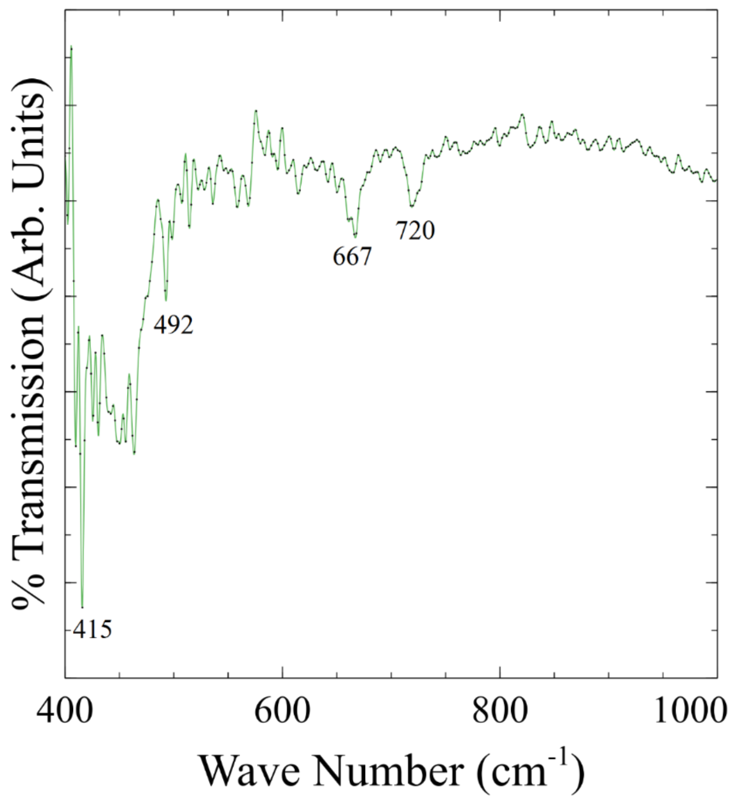

Fig. S3. Fourier transform infrared (FTIR) spectra collected on a slice of a PZT stack.

Previous literature (26) shows the optical vibrational phonon mode frequency at 410 cm^{-1} for bulk PZT and 420 cm^{-1} for thin films; here, we measure the frequency to be 415 cm^{-1}. The variations are due to the different composition of this PZT. The measurement was taken on a Perkin Elmer Frontier Dual Range FIR/MidIR spectrometer.

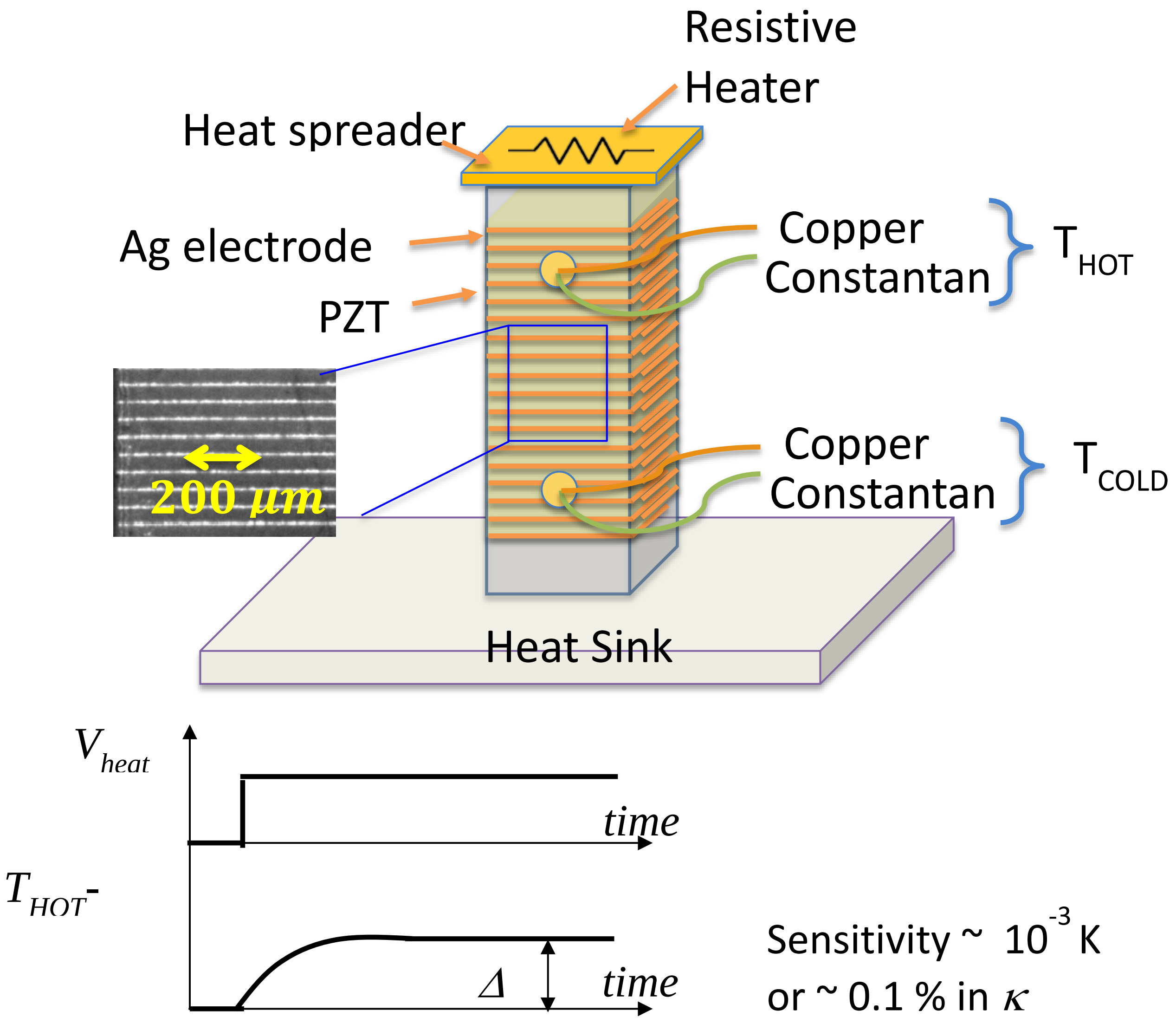

Fig. S4. Static heater and sink method. The sample is equipped with a resistive heater and mounted on a heat sink (see methods). After current is applied to the heater, the temperature difference on the sample is allowed to equilibrate and is then measured.

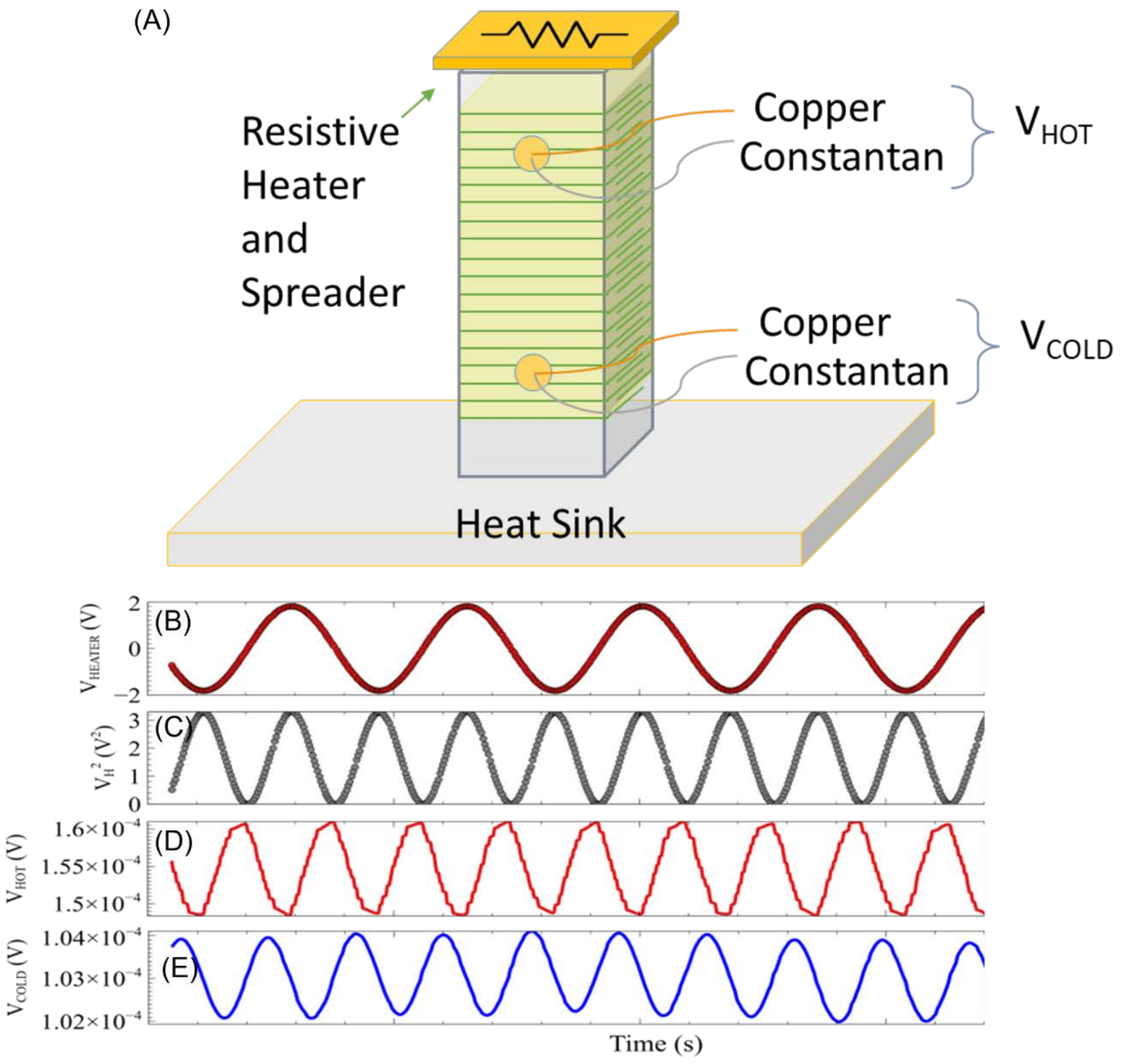

Fig. S5. Ångström method

(**A**) The same sample mount is the same as for the static method (Fig. S4), but a sinusoidal voltage (**B**) is applied to the heater. This produces the heating power time dependence shown in (**C**). The measured voltages on the two thermocouples are shown in (**D**) and (**E**). The attenuation and phase shift between these two signals are used to derive the diffusivity as explained in the methods section. We further assume that the two thermocouples have the same calibration, so that the voltages from the thermocouples, V_{HOT} and V_{COLD} are used in the data treatment instead of the temperatures T_{HOT} and T_{COLD}.

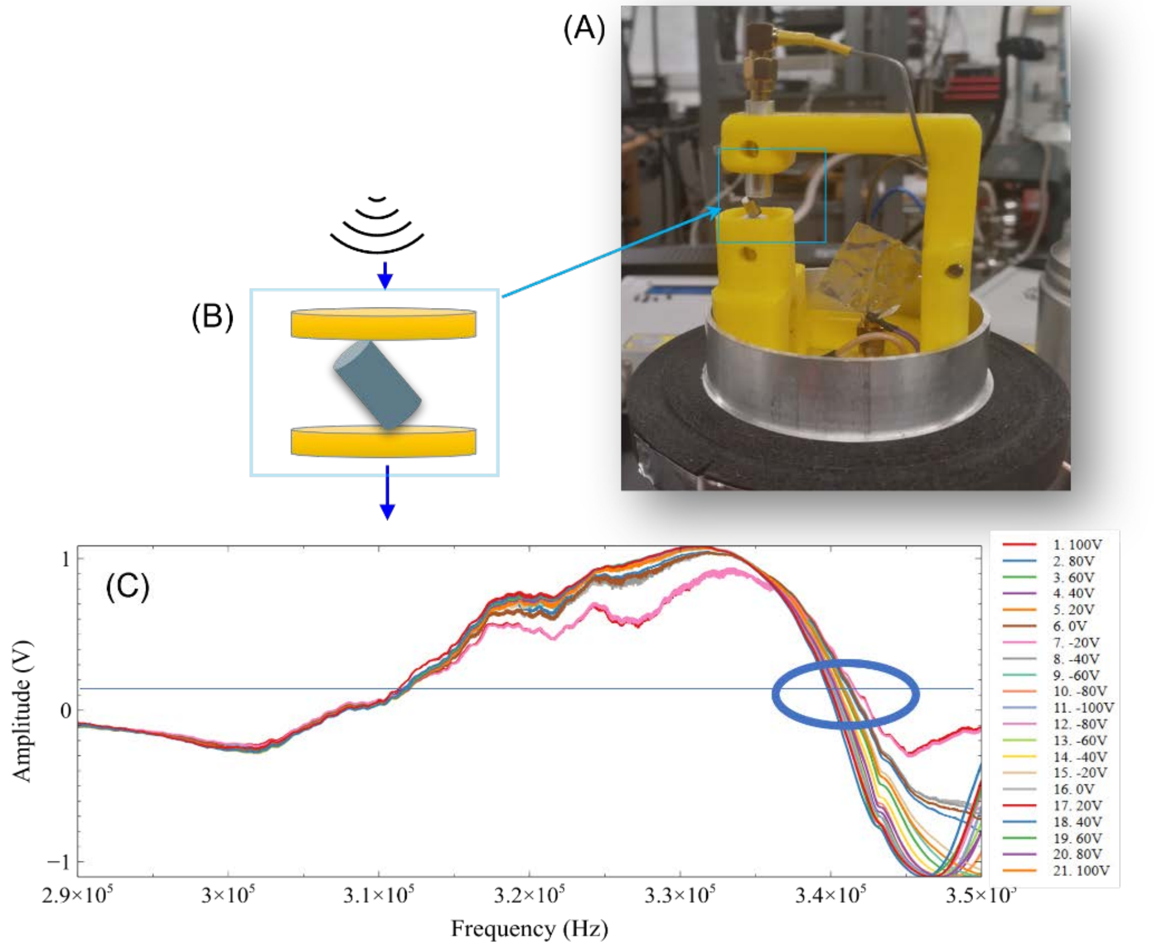

Fig. S6. Resonant ultrasound spectroscopy (RUS)

(**A**) A picture of the RUS instrument. (**B**) shows a schematic of the highlighted portion. The sample (grey cylinder) is clamped between two ultrasound transducers (yellow plates) and a variable frequency excitation is applied to one of the transducers, while the voltage on the other is recorded, giving the amplitude of the vibrations. The in-phase and out-of-phase components of the amplitude of the vibration are plotted as function of frequency. (**C**) shows the out of phase results where a zero crossing indicates a resonant mode, emphasized with the blue circle. In this experiment, one particular frequency around 340 kHz is identified with the longitudinal compressive resonant mode of the stack (blue circle), and its shift is measured as a function of the voltage applied to the sample (see methods section).

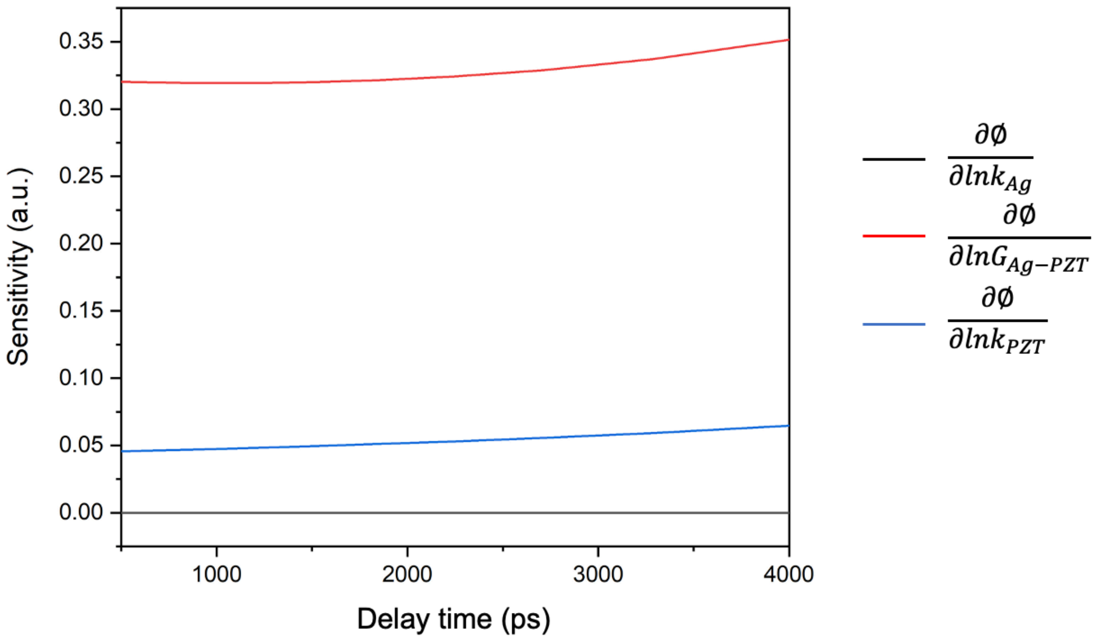

Fig. S7 Sensitivity of the TDTR measurements.
TDTR measurement sensitivity as a function of the pump-probe delay time. Black, red, and blue lines indicate measurement sensitivity for Ag thermal conductivity k_{Ag}, interfacial thermal resistance $G_{Ag\text{-}PZT}$ between Ag and PZT, and thermal conductivity of PZT, k_{PZT}, respectively.
